# Latitudinal gradient of thermal safety margin in an Australian damselfly: implications for population vulnerability

**DOI:** 10.1098/rsos.241765

**Published:** 2025-03-05

**Authors:** Md Tangigul Haque, Shatabdi Paul, Marie E. Herberstein, Md Kawsar Khan

**Affiliations:** ^1^ School of Natural Sciences, Macquarie University, North Ryde 2109, Australia; ^2^ Department of Biology,Chemistry and Pharmacy, Freie Universität Berlin, Berlin 14195, Germany

**Keywords:** climate change, insect decline, parasite, critical thermal maximum, thermal safety margin, disease ecology

## Abstract

The thermal tolerance of species may be exceeded by the predicted temperature increases and thus contribute to species extinction. However, the impact of temperature increases is thought to vary between climate regions and across latitudes. Here, we aim to establish the vulnerability of an ectothermic insect to a warming climate by estimating the thermal safety margin in *Ischnura heterosticta* damselflies. We measured the critical thermal maximum (CTmax) along a latitudinal gradient of 17° from 21 populations along the eastern coast of Australia. Our results showed that damselflies inhabiting tropical regions had higher CTmax than temperate damselflies. CTmax increased with increasing mean temperature and decreasing latitude. We further found a positive correlation between damselfly parasite number and temperature. Body size, body condition and sex had no impact on CTmax. Our projections showed that the damselfly thermal safety margin will be narrower in the tropics compared with temperate regions under a predicted 2.6°C annual mean temperature (future projected – current) increase for the years 2061–2080. Therefore, damselflies in the tropics are likely to be more vulnerable to climate change-driven extinction even though they have a relatively higher CTmax. Nevertheless, behaviour, temperature adaptation and thermal plasticity might mitigate predicted vulnerability.

## 1. Introduction

Some organisms experience narrow and limited temperature fluctuations, while others experience significant variation in ambient temperature across space, largely shaped by local climatic factors, which determine the thermal ranges of species and populations across altitude and latitude [1,2]. In general, species inhabiting lower latitudes and particularly regions near the Equator experience a relatively stable thermal environment due to lower seasonal variation in temperature [3]. Higher latitudes, on the other hand, are associated with more variable temperatures [3]. As a result, populations in tropical and lower latitude regions are generally thought to have a lower thermal range than species in higher latitude regions [3]. Over the last decades, anthropogenic activity has been increasing global temperatures and the frequency of extreme heatwaves, thereby posing significant challenges for organisms to adapt to the rapidly changing climatic conditions [4,5]. Understanding the thermal range of populations across latitudes can help to determine the climate resilience of populations and species [6]. Broader knowledge on the thermal range of species is required for developing effective management strategies to conserve biodiversity in a warmer world.

Arthropods are thermally vulnerable, especially at the warmer edges of their distribution, under increasing global temperatures [7]. Thermal tolerance describes the range of temperatures at which organisms can perform their physiological functions. The critical thermal maximum (CTmax) of organisms often indicates how they might fare under projected warmer climates and increased heatwaves [8,9]. The CTmax can vary intra- and inter-specifically [10,11], across seasons [11,12] and even along urban–rural gradients [13].

Abiotic climatic factors, body size (e.g. thorax size), physiology and infection can impact CTmax and therefore the thermal safety margin (CTmax minus the maximum temperature of the warmest month) [14,15]. For example, smaller and paler butterfly species have higher thermal tolerance than larger and darker butterflies [16]. Smaller insects with higher surface area to volume ratio (SA : V) have excess water loss rates which help them to cope with high environmental temperatures by facilitating heat dissipation [17]. However, this strategy is not always successful as higher surface area to volume ratios (SA : V) in small ectotherms lead to faster heat transfer rates and increased desiccation risk [18]. In addition, infections with parasites, bacterial, fungal and viral pathogens might also impact CTmax and hence thermal safety margin [19–21]. For example, bacterial (*Pasteuria ramosa*) infection causes a reduction of upper thermal limits in water fleas (*Daphnia magna*) [21]. However, pathogens can also boost host thermal tolerance by activating common pathways (such as Imd, Toll and molecular chaperones) that are also involved in thermal stress response ([20] and references therein). This host–pathogen response to thermal stress is context-dependent, and there is very little or no evidence available about the exact mechanisms. For example, desert locust *Schistocerca gregaria* when infected with the fungus *Metarhizium anisopliae*, can tolerate higher temperatures (up to 44°C) than their optimal ambient temperature (40°C) [22,23].

Insects experience variation in microclimate across different life stages, concomitant with different physiological adaptations to respond to temperature and other environmental factors [24–26]. For example, butterfly and bee larvae reared in different environmental conditions have the capacity to adapt to local climate via shifting their optimal and maximum temperature range [27,28]. Specifically, solitary bee larvae (*Osmia ribifloris*) were reared in previous cooler (1950) and projected future warmer (2040−2099) conditions and responded to the higher temperatures by emerging later with greater variation in emergence time than in cooler temperatures [27]. Moreover, the larval environment can also impact adult fitness—for example, butterfly late-stage larvae exposed to high temperatures have a reduced reproductive output as adults [28]. Similarly, most aquatic insects typically spend their immature stages in the water and emerge onto land as adults [29]. Water, as a medium, has greater thermal buffering abilities than air [30]. This leads to water temperatures being more stable and less prone to fluctuation when compared with air. This may mean that aquatic larvae are more protected from short-term temperature fluctuations. However, when water temperatures actually increase, this may lead to faster growth rates (until an optimum is reached), shorter development times and smaller size at emergence [29].

Odonates (damselflies and dragonflies; Insecta: Odonata) are semi-aquatic ectotherms with an aquatic larval stage and a terrestrial adult stage. The temperature experienced by larval odonates has been shown to impact their growth, survivability and thermal limits [31,32]. For example, dragonfly nymphs inhabiting warmer streams have a higher thermal tolerance than those in cooler streams [33]. In addition, a common-garden experiment on larval *Ischnura elegans* damselflies showed that individuals from low-latitude regions can tolerate higher temperatures than high-latitude individuals [34]. Another study found that dragonfly larvae (*Sympetrum vulgatum*) reared at high temperature showed higher growth rates and mortality rates; however, temperature had no impact on growth and survival of larvae from a different species within the same genus, *Sympetrum fonscolombii* [31].

The impact of climate, especially temperature, on the thermal tolerance of adult odonates determines how odonates might perform physiological functions under rapidly changing climates, yet it has been less of a focus in the literature. Thus, the aim of our study is to understand how temperature might impact damselfly populations by establishing the thermal safety margin in the damselfly, *Ischnura heterosticta* (Burmeister, 1839) (Coenagrionidae), across different biomes (temperate, subtropical and tropical). In our study, we tested the climate variability hypothesis: individuals living in thermally stable environments are predicted to have a narrow thermal tolerance [35,36] and hence are more vulnerable at forecasted future temperatures. Based on this hypothesis, we predicted that tropical damselflies have higher CTmax (increases with decreasing latitude) but a narrower thermal safety margin—being more vulnerable in a rapidly changing climate. In addition, physiological stress due to parasitization may result in a lower CTmax, thereby narrowing the thermal safety margin. To estimate the thermal safety margin in current and future climates, we measured CTmax for a large number of populations across a 2700 km gradient. We analysed how CTmax varies with intrinsic (size, sex and parasite load) and extrinsic (latitude and local climate) factors. We further calculated the thermal safety margin and compared the three major biomes.

## 2. Material and methods

### 2.1. Study species

*Ischnura heterosticta* is a damselfly with an average body size of 33.7 ± 0.08 mm. They are widely distributed throughout Australia, including Tasmania, and commonly found near stagnant or slow-moving water reservoirs such as ponds, lakes, marshes and lagoons [37]. Their high tolerance to salinity also allows them to exploit brackish water [38,39]. Adult males are identified by a bright blue thorax and blue abdominal stripes on segments 8 and 9. Females undergo colour change during ontogeny: the pre-reproductive andromorph females have a blue thorax and blue abdominal stripes on segments 8 and 9, whereas adult heteromorph females have a grey thorax and abdomen [40]. Usually, *I. heterosticta* can be seen throughout the year; however, their flight activity peaks between October and March [40].

### 2.2. Study sites

**Figure 1.**
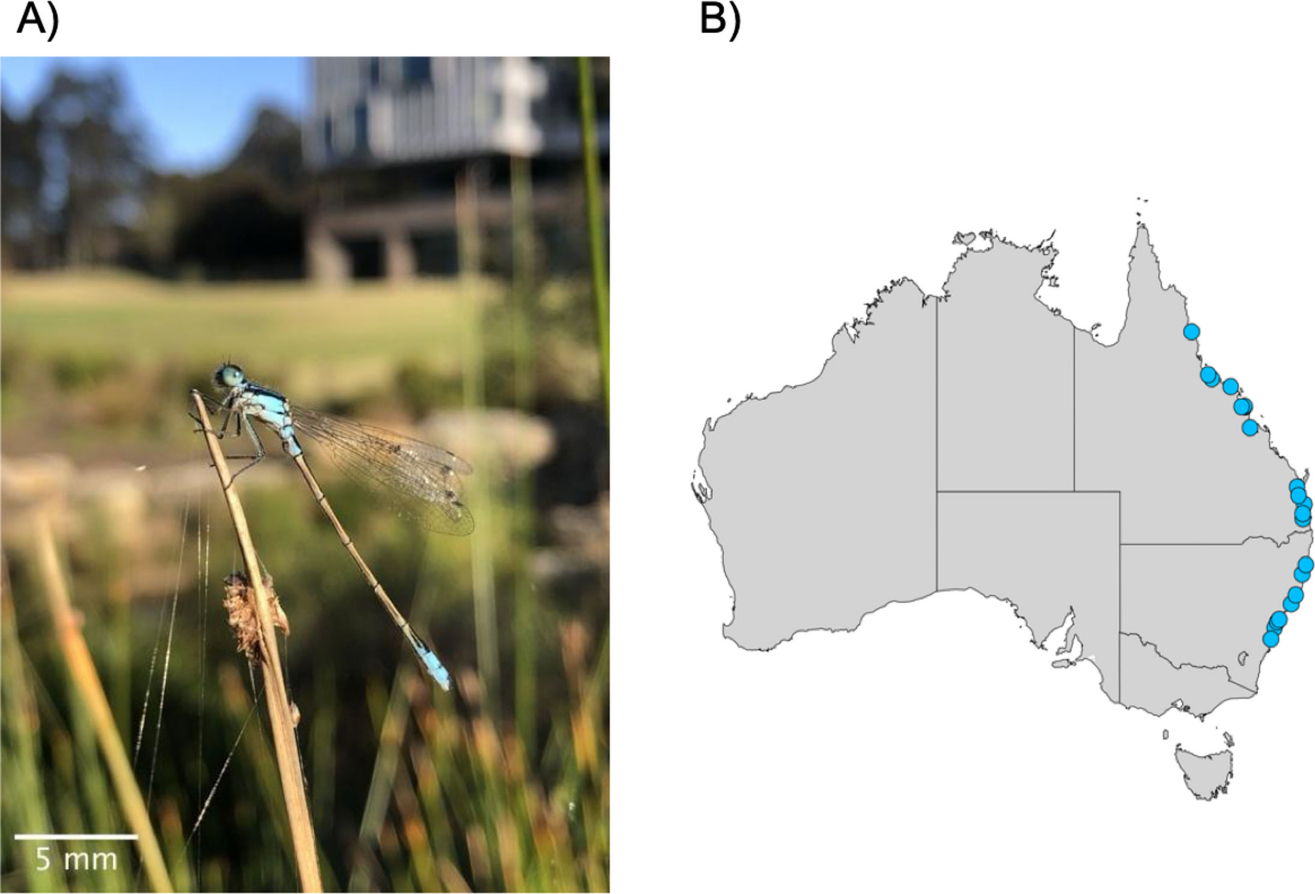
(A) Image of male *I. heterosticta* at Macquarie University, Sydney, Australia. Photo © Shatabdi Paul. (B) Sampling sites—circles represent different field sites along the east coast of Australia, map created in R using the exact sampling location.

We collected *I. heterosticta* from 21 populations along the East Coast of Australia—from Cairns (16°52′1.2″ S, 145°41′6″ E) to Wollongong (34°24′43.2″ S, 150°52′40.8″ E), covering a total of approximately 2700 km and 17.6° (16.8° S–34.4° S) latitudes (figure 1). We collected damselflies from 23 September to 31 October 2022. The average distance between field sites was approximately 113.32 ± 5.65 km. We collected damselflies during the early spring because the weather conditions were suitable for their activity and they were high in abundance: many species emerge from the nymph stage, transition to adult stage and actively fly and mate during this time.

### 2.3. Damselfly collection

Damselflies were captured with an insect sweep net (dimensions: 1260 mm handle, 456 mm diameter hoop, 456 mm diameter polyester bag) between 09.00 and 16.00 hours when they were most active in the field. We then transported live damselflies from the field to the experimental set-up in mesh travel containers (diameter: 14 cm, height: 23 cm (expanded), 1.5 cm (packed)). During transportation, damselflies were sprayed with water to keep them hydrated. Before starting the thermal tolerance experiment, we recorded the sex and parasitism status (whether parasites were present and, if so, the number of parasites: water mite*—Arrenurus*) of each damselfly. We conducted a thermal tolerance experiment within approximately 4 h after collecting damselflies from the field sites. We kept damselflies at 25°C temperature and 70% relative humidity prior to conducting the experiment. No permits were required to collect the specimens as *I. heterosticta* is not endangered in Australia and the study sites were not located in protected areas or national parks.

### 2.4. Critical thermal maxima

CTmax is the upper temperature limit and critical thermal minimum (CTmin) is the lower temperature limit [41,42]. CTmax represents the temperature when organisms lose motor function and perform uncoordinated movement [43]. We used a dynamic ramping method (gradually heating up the animals) to measure the CTmax of damselflies. Before starting the experiment, we checked whether damselflies were alive, active and did not have wounds or damaged wings. We placed damselflies in a 15 ml centrifuge tube (Sarstedt AG & Co. KG, Nümbrecht, Germany) and submerged them in a water bath (model: MyBath™ mini water bath, Benchmark Scientific B2000-4-T5; accuracy: ±0.5°C). An external thermometer placed into the water bath was used to ensure temperature accuracy. We kept damselflies in the water bath at 25°C for 15 min and allowed them to acclimatize. We chose the constant acclimatization temperature for all populations because we collected damselflies during similar weather conditions: sunny and warm days with temperatures ranging from 20°C to 30°C. Next, we increased the water bath temperature by 1°C with an average ramp rate of 0.38°C min^−1^ which took on average 2.6 min (± 0.25 s.e.) and kept the damselflies at this temperature for 3 min. We checked whether the damselflies were active at this new temperature. We continued this process, increasing the temperature by 1°C at a time, until damselflies were knocked down. We recorded the temperature at which damselflies lost their coordinated movement. At the completion of the experiment, we placed them in 95% ethanol and stored them at −30°C for further measurements [44]. We used the DurgaDiff function of the Durga R package to quantify the difference in thermal tolerance across tropical, subtropical and temperate regions [45].

We did not consider the impact of colour change on damselflies CTmax, as our experimental design did not involve irradiation. The water bath transfers heat through conduction and convection, and not via incoming light, hence there was no colour-related absorption or reflection during our experiment. However, we discuss the potential impact of colour on damselfly thermoregulation in a later section.

### 2.5. Body condition measurement

Damselflies were removed from the ethanol and placed on absorbent paper for exactly 2 min to evaporate the ethanol [46]. We measured the body weight of each damselfly using a Mettler Toledo analytical balance (model ML204T/00, accuracy 0.0001 g). Next, we placed the damselflies in a lateral position and photographed each damselfly using a Canon EF 50 mm f/2.5 compact macro lens against a white background with a scale bar. We used Adobe Photoshop software v. 6.0 to prepare the layout of the photographs. We then measured the body length of damselflies using ImageJ software (v. 1.53) [47]. We calculated the body condition of damselflies using the scaled mass index (SMI) method by applying the formula


$$M_{i}=M\times (L_{0}/L{)}_{SMA}^{b}.$$

Here, *M* and *L* are the body mass and body length of the individual, respectively. *L*_0_ is the mean of *L* and *^b^*_SMA_ is the scaling component estimated by the standard major axis (SMA) regression of log-transformed body mass on log-transformed body length [48]. The SMI method is considered the most appropriate and widely used method for calculating the body condition of an organism [48,49]. We applied the DurgaDiff function of the Durga R package to determine whether body size and weight vary between sexes [45].

### 2.6. Climatic variables

Latitude and climatic factors such as temperature may influence insect thermal tolerance. We therefore extracted the daily maximum temperature (°C) of sampling sites from the Australian Government Bureau of Meteorology (BOM) (http://www.bom.gov.au). We downloaded monthly (October–March) average temperature (°C) data using our field site coordinates from the WorldClim database v. 2.1 (https://www.worldclim.org) with a spatial resolution of 30 s [50]. We selected these months because they capture the activity periods of *I. heterosticta* (Khan, personal observation). We used the R package ‘raster’ to extract climate data from the database [51]. We used the ‘corr’ function in R to determine the correlation between daily maximum temperature (°C) and monthly mean ambient temperature (°C) [52]. We performed principal component analysis (PCA) between these highly correlated (*r* = 0.78) climatic variables and selected PC1 that explained 89.5% variance in the data for further analysis. Both daily maximum temperature (°C) and monthly mean ambient temperature (°C) were positively correlated with PC1.

To determine what climatic factors and physiological conditions explain the variation of thermal tolerance, we applied generalized linear mixed models using template model builder (glmmTMB) [53]. We fitted models with CTmax as a response variable, PC1, body size, body condition, parasite numbers and sex as fixed factors, and study site as a random factor. PC1 and latitude are highly correlated (*r* = 0.93). We therefore built a separate model with CTmax as a response variable, latitude as a fixed factor and study site as a random factor to determine whether thermal tolerance varies with latitude. We then checked residual plots and goodness-of-fits of models using the DHARMa package [54]. All analyses were conducted in R v. 4.1.2 [52]. All values are mean ± s.e.

### 2.7. Estimating current and future thermal safety margin

We extracted historical (1970−2000) and future (2061−2080) climatic data (maximum temperature of the warmest month (BIO5) from the WorldClim database at 10 min spatial resolution). Then, we measured historical/near current and future thermal safety margin by subtracting the value of maximum temperature of the warmest month from CTmax of each damselfly.

## 3. Results

**Figure 2.**
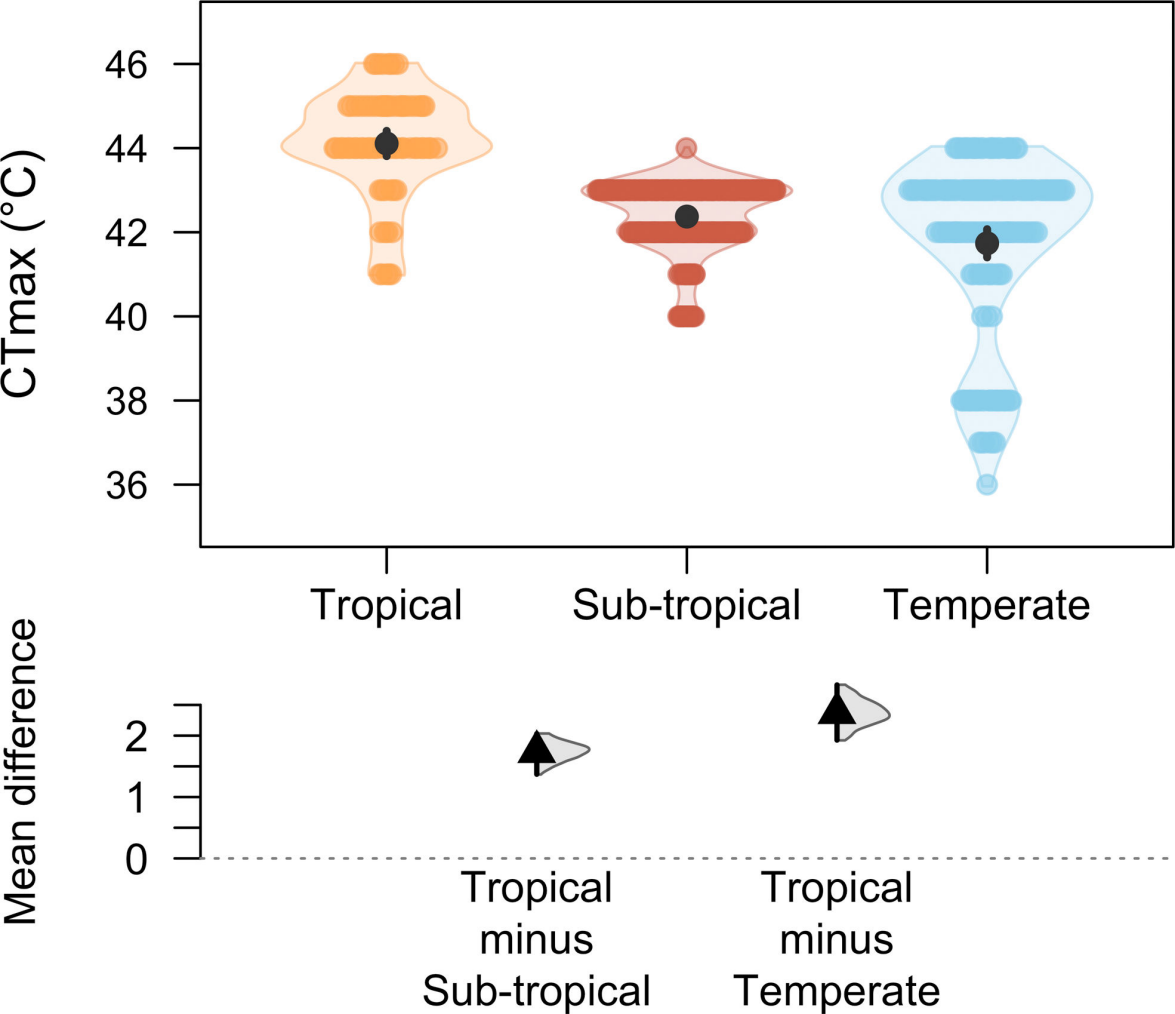
DurgaPlot [45] shows large mean differences in CTmax from tropical to temperate regions and moderate mean differences in CTmax from tropical to subtropical regions. Orange, coral and blue represent tropical, subtropical and temperate regions, respectively. The black circle represents the mean and the vertical bar represents the confidence intervals while the coloured dots represent the CTmax of an individual damselfly. The black triangle represents the mean difference, the vertical line represents the 95% CI of the mean difference and the half violin represents the density of the bootstrapped mean difference.

We captured a total of 433 damselflies (216 males and 217 females) from 21 different field sites. The number of damselflies in each site varied based on their availability, ranging from a minimum of 14 to a maximum of 33 individuals. Our data provided no evidence that body length differs between sexes (female body length: 33.8 ± 0.12 mm, male body length: 33.7 ± 0.12 mm; mean difference: 0.045, 95% CI [−0.30, 0.38]). However, the body mass of females (39.6 ± 0.53 mg) was greater than that of males (31.2 ± 0.34 mg) (mean difference: 8.389, 95% CI [7.17, 9.67]). The average CTmax of the damselflies, pooling all sites, was 42.4 ± 0.08°C, ranging from 36°C to 46°C. The highest CTmax was in tropical populations and the lowest was in temperate populations (figure 2).

**Figure 3.**
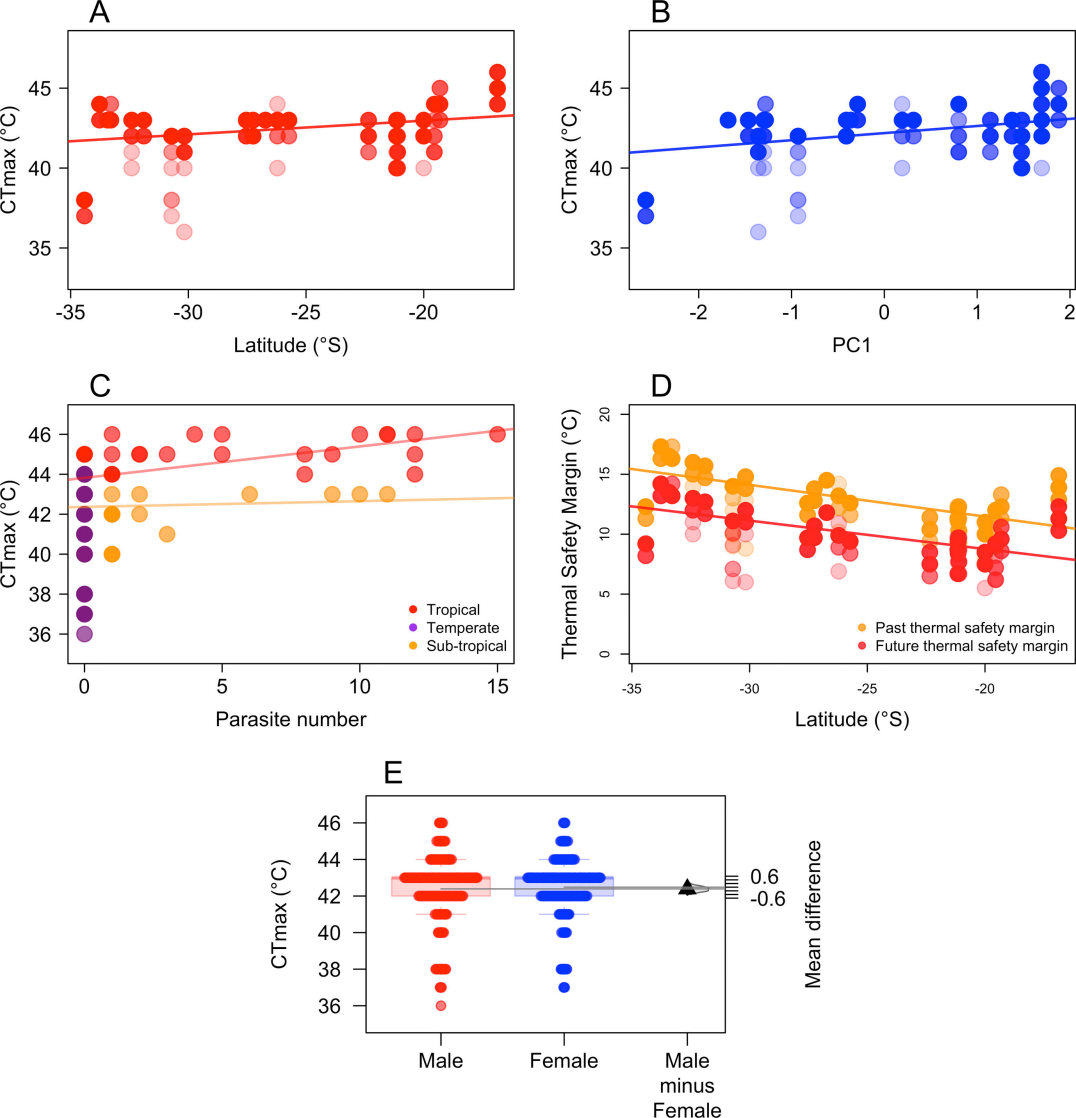
(A) CTmax of *I. heterosticta* slightly increases with decreasing latitudes; (B) CTmax increases with PC1 (includes daily maximum temperature (°C) and monthly mean ambient temperature (°C), which are positively correlated with PC1), representing higher CTmax at high daily maximum temperature (°C) and mean annual temperature (°C); (C) parasite number increases with CTmax. There is no regression line in temperate regions as temperate individuals had no parasites; (D) thermal safety margin is predicted to decrease with latitude under future climate change, showing a narrowing of the thermal safety margin in the tropics; coloured circles in panels A–C (dark colour denotes many individuals have the same CTmax where light colour indicates fewer individuals have the same CTmax) represent CTmax of an individual damselfly. The solid lines represent linear regression of the response variable (CTmax) with the various predictors. (E) CTmax is lower in males than females, coloured circles represent CTmax of an individual damselfly. The black triangle represents the mean difference, the vertical line represents the 95% CI of the mean difference and the half violin represents the density of the bootstrapped mean difference.

There was weak evidence that CTmax increased with decreasing latitudes (glmmTMB: estimate = 0.08 ± 0.05, *Z* = 1.70, *p* = 0.089, figure 3A). Additionally, CTmax was positively correlated with PC1 (glmmTMB: estimate: 0.44 ± 0.20, Z = 2.19, *p* = 0.028, figure 3B) and parasite number (glmmTMB: estimate: 0.05 ± 0.02, *Z* = 2.37, *p* = 0.017, figure 3C) but not with body size (glmmTMB, estimate: 0.01 ± 0.02, *Z* = 0.58, *p* = 0.564), body condition (glmmTMB, estimate: −6.86 ± 9.74, *Z* = −0.70, *p* = 0.480). The effect of CTmax on parasite load was, however, climate-dependent and CTmax increased with parasite load in the tropics but not in the subtropical and temperate regions (figure 3C). Our results also showed that males had a slightly lower CTmax than females (estimate: −0.19 ± 0.11, *Z* = −1.67, *p* = 0.095, figure 3E), although the evidence was weak.

**Figure 4.**
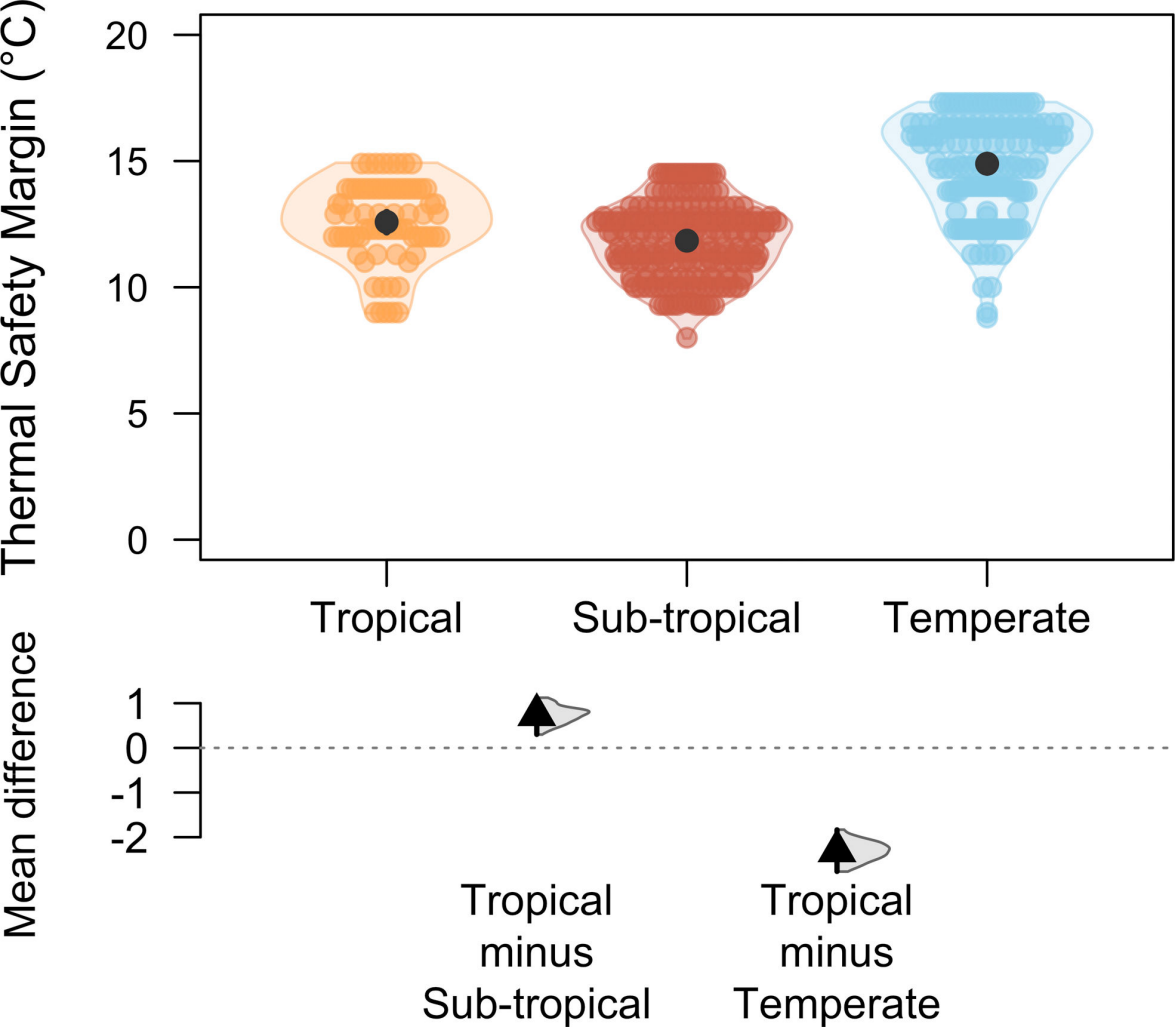
DurgaPlot [45] showing thermal safety margins will be lower in both tropical and subtropical regions compared with temperate regions under future climates due to temperature rise. Orange, coral and blue represent tropical, subtropical and temperate regions, respectively. The black circle represents the mean and the vertical bar represents the confidence intervals while the coloured dots represent the thermal safety margin. The black triangle represents the mean difference, the vertical line represents the 95% CI of the mean difference and the half violin represents the density of the bootstrapped mean difference.

Analysing climate forecast data, we showed that the thermal safety margin of damselflies decreased with decreasing latitudes (glmmTMB, estimate: −0.23 ± 0.05, *Z* = −4.22, *p* < 0.001, figure 3D) and tropical populations had a narrower thermal safety margin than temperate populations (mean difference: −2.30, 95% CI [−2.80, −1.84]; figure 4).

## 4. Discussion

The aim of our study was to quantify CTmax of *I. heterosticta* damselflies to estimate the thermal safety margin of these odonates under predicted temperature increase. As thermal tolerance is dependent on a complex interplay of internal and external factors, we explored the impact of sex, body size, body condition, the presence of parasites and ambient temperature on damselflies CTmax along a latitudinal gradient. We found CTmax varies in different climatic regions and was highest in the tropics, followed by subtropics and temperate regions in support of the climate variability hypothesis. However, we observed only a weak correlation between CTmax and latitude, while CTmax was positively correlated with daily maximum temperature, annual mean temperature and parasite load.

We hypothesized that tropical damselflies will be more vulnerable than temperate damselflies due to global warming, though they have higher CTmax. Our results provide evidence that the thermal safety margin is narrowing in the tropics, with the potential of increasing the threat to tropical damselflies as global temperatures increase. Our result is consistent with previous studies that found a decrease of CTmax with increasing latitude (i.e. away from the Equator) [15,55] and local temperature [15,35]. Lower latitudes have comparatively higher temperatures, and tropical insects are locally adapted to high temperatures [56,57]. Moreover, other factors (such as precipitation, humidity and elevation) can modify the impact of temperature on CTmax, resulting in complex multi-factorial patterns across different biomes [6,58]. Furthermore, insects modify behavioural and physiological functions [59,60] to adapt to higher temperatures.

Insects evolved both plastic and genetic responses to temperature fluctuations and extremes [61,62]. The high CTmax we observed in tropical regions may be due to physiological plasticity [15,63] resulting in a short-term increase of CTmax when challenged with higher ambient temperature by reducing metabolic rate or by inducing heat shock proteins. For example, tropical bees (*Apis melifera jemenetica*) have a higher expression of heat shock proteins (e.g. hsp10, hsp28, hsp70ab, hsp83 and hsp90 mRNAs) during short-term temperature increases. These proteins are thought to protect the bees at high temperatures during the summer season [64]. Epigenetic effects can also contribute to higher CTmax in tropical populations, whereby experiencing heat stress in one generation can influence the heat tolerance in the next generation [65]. Evidence for genetic change in response to selection via temperature extremes is highly desired but is scarce for wild populations but has been established in laboratory models such as *Drosophila* [66]. Finally, damselfly behavioural thermoregulation may reduce exposure to high temperatures, such as (i) moving to cooler microhabitats (shaded areas) or (ii) changing activity—becoming less active during the hotter time of the day. Furthermore, thermal buffering, the ability to maintain body temperatures across a range of ambient conditions [16]; may allow damselflies to cope with temperature stress, which is often inversely related to CTmax.

Our model suggested that parasite load increased CTmax in the host damselfly. While it is possible that parasite infection could induce the expression of heat shock proteins, which ultimately increase CTmax [67], in our study, it is more likely that these statistical results are an artefact of the relationship between parasite load and temperature (PC1), which also correlates with CTmax. We further tested this using a model identifying interaction between temperature and parasite load (electronic supplementary material, S1). Our results suggest that the relationship between parasite number and CTmax is likely to be directly influenced by temperature, which reflects variation across different climatic regions: tropical, subtropical and temperate. In tropical populations, CTmax increased with parasite load; however, this trend was absent in subtropical and temperate regions. The lower parasite load in the subtropical and temperate regions is limiting the strength of the analysis, and further targeted sampling of parasitized and non-parasitized damselflies from populations across climatic zones could help us to better understand the impact of parasites on CTmax.

Colour in odonates may be associated with thermal tolerance, as species at higher latitudes are often darker than at lower latitudes [68,69], which is thought to provide a thermal advantage whereby darker individuals at higher latitudes absorb more irradiation than the lighter individuals at lower latitudes. However, it is unclear to what extent colour influences thermal tolerance. For example, there is contradictory evidence on how wing pigmentation in damselflies (e.g. *Calopteryx*) relates to thermal tolerance [70,71]. Moreover, thermal modelling of colour-changing phenotypes (e.g. dark brown and turquoise grasshoppers) indicates little, if any, thermal benefit from colour change [72].

Our overall aim was to estimate the thermal safety margin of *I. heterosticta* in order to predict how vulnerable odonates like *I. heterosticta* might be in a warming world. Similar to previous studies on ectothermic insects and amphibian tadpoles [59,73,74], we found a narrower thermal safety margin in the tropics compared with subtropical and temperate regions. This suggests that tropical populations of damselflies may be more vulnerable to anthropogenic global warming. While tropical species usually experience warmer temperatures throughout the year compared with temperate species, their optimal temperature [75,76] is close to their CTmax [3]. So, even small increases in temperature may reach or even exceed their thermal tolerance [77]. It might be possible that species gradually shift their CTmax through plastic response to thermal stress (sometimes with limited capacity); however, they may struggle to effectively mitigate the response to heatwaves, which are predicted to increase [78–80]. Recently, a study found that heatwaves increased the mortality rate of solitary bee larvae by 130% (*Osmia lignaria*; [81]). The baseline temperature of heatwaves in Australia is greater than 40°C, which exceeds some of the CTmax we recorded in *I. heterosticta* individuals. The 2024 highest daily maximum temperature in New South Wales (nearest to our field sites), Australia, peaked at 45.4°C (http://www.bom.gov.au), which exceeded almost all of our CTmax estimates in *I. heterosticta* damselflies.

The relationship between biome and thermal safety margin might not be linear, with subtropical species being more vulnerable than tropical or temperate ones [82]. In addition, Johansson *et al.* [83] found that temperate species with narrower activity periods were as vulnerable as tropical species [83]. It is also possible that a faster warming rate in temperate regions increases vulnerability to climate change even though temperate populations experience greater climate variation [84]. Clearly, more studies are required—both within and across generations—including large-scale studies in the Southern Hemisphere to further understand the general pattern of species vulnerability [85].

One of the limitations of our study is that we used estimated ambient temperature, maximum temperature of the warmest month, in our study, which possibly does not capture the actual temperature that adult damselflies experience in natural habitats. Moreover, we were unable to incorporate heating effects of solar radiation in our experiment, which may vary across gradients due to varied integument pigmentation of damselflies across latitudinal gradient. More studies are required focusing on both radiative heat effects and field-based measurement of actual body temperature to accurately predict the impact of climate change on damselfly thermal tolerance.

In summary, our study showed that CTmax is higher closer to the Equator; however, the thermal safety margin is narrower compared with higher latitudes. The vulnerability due to a narrower thermal safety margin is independent of intrinsic factors (sex, size and condition) but whether the vulnerability might be mitigated by plastic and evolutionary responses should be the focus of future research.

## 5. Statement of diversity inclusion

We strongly support equity, diversity and inclusion in science [86]. The authors come from different countries (Bangladesh, Austria and Australia) and represent different career stages (PhD candidate, Early Career Researcher and Professor). One or more of the authors self-identifies as a member of the LGBTQI+ community. One or more authors are from an underrepresented ethnic minority in science.

## Data Availability

All data and code is deposited in Figshare [87].
